# Identification and Biological Features of Sesame Phyllody-Associated Phytoplasmas in Western Iran

**DOI:** 10.3390/pathogens15030339

**Published:** 2026-03-23

**Authors:** Samira Pakbaz, Ehsan Hasanvand, Assunta Bertaccini, Sara Gharouni-Kardani

**Affiliations:** 1 Department of Plant Protection, Faculty of Agriculture, Lorestan University, Khorramabad 6815144316, Iran; 2 Qazvin Agricultural and Natural Resources Research and Education Center, Agricultural Research, Education and Extension Organization (AREEO), Qazvin P.O. Box 19395-1113, Iran; e.hasanvand@areeo.ac.ir; 3 *Alma Mater Studiorum*, University of Bologna, 40127 Bologna, Italy; 4 Department of Plant Protection Research, Khorasan Razavi Agricultural and Natural Resources Research and Education Center (AREEO), Mashhad P.O. Box 19395-1113, Iran; s.gharooni@areeo.ac.ir

**Keywords:** *Sesamum indicum*, phytoplasmas, PCR/RFLP analyses, sequencing, dodder transmission

## Abstract

Sesame is an oilseed crop threatened by a phyllody disease associated with the presence of phytoplasmas, which can reduce yields by up to 80%. The molecular identification of these bacteria in crops located in Western Iran was achieved from samples showing symptoms of diverse intensity and types. For biological characterization, the pathogen was also dodder-transmitted to periwinkle plants. After nucleic acid extraction and nested PCR using phytoplasma-specific primer pairs amplifying part of the 16S rRNA gene, it was possible to amplify DNA fragments from both symptomatic sesame samples and dodder-inoculated periwinkle plants. The virtual RFLP pattern from the 16S rRNA gene sequences using *i*PhyClassifier indicated the presence of phytoplasma strains in 16SrII-D and 16SrIX-C subgroups. The identity percentage values of the obtained amplified sequences corroborated by the phylogenetic analysis identified them as ‘*Candidatus* Phytoplasma australasiaticum’ and ‘*Ca*. P. phoenicium’, respectively. The two phytoplasma strains were detected in different sesame samples collected in the same field. The coexistence of two phytoplasmas may influence the observed differences in disease severity and suggests appropriate management strategies, since diverse insect vectors were reported alongside diverse phytoplasmas associated with this disease. Moreover, the widespread disease presence strongly suggests the breeding of resistant varieties.

## 1. Introduction

Sesame (*Sesamum indicum* L.) is one of the oldest oil crops grown in many parts of the world [1]. Its seeds are a rich source of oil (about 50%) and protein (approximately 20%) [2]. Sesame seed oil has a long shelf life due to the presence of the antioxidant sesamol and also has a significant amount of oleic acid. It is used for stir-frying, dressings, and as a finishing oil in cuisine, as well as industrial material for producing paints, varnishes, soaps, perfumes, pharmaceuticals, and insecticides. Sesame has traditionally been cultivated in the hot and dry regions of Iran, where its low water requirement has contributed to its cultivation and increased its popularity [3]. Currently, sesame ranks second in cultivated area among oilseed crops, following rapeseed, and its cultivation is widespread in over 20 of Iran’s 31 provinces, including Lorestan. According to statistics, the sesame yield in Iran reached 929 kg/ha in 2022 [4]. From 2000 to 2022, the average sesame yield in Iran exceeded the global average [3]. However, sesame is susceptible to infection by various pathogens, which can lead to significant yield losses. Phyllody is considered a very severe disease, inflicting in areas such as India up to 80% crop loss, and affecting between 61% to 80% of the plants in a single field [5]. Sesame phyllody disease was first recorded in British India, now Pakistan, in Mirpur Khas in 1908 [6], where it has been associated with the presence of a phytoplasma belonging to subgroup 16SrII-D [7]. Phytoplasmas are wall-less, phloem-inhabiting bacteria within the class *Mollicutes*. They are associated with severe damage to crops and are known to infect around 1000 plant species worldwide, including fruits, vegetables, cereals, trees, and legumes. The most common symptom associated with the presence of phytoplasmas in sesame is phyllody, which consists of flower virescence and proliferation, and abnormal leaf growth sometimes accompanied by yellowing, cracking of seed capsules, seed germination within capsules, and witches’ broom [8]. Although phytoplasma has been reported in sesame seeds and seedlings [9], the disease is mainly spread in the fields in Iran by the leafhopper *Orosius albicinctus* [10], and the associated pathogen survives in alternate hosts such as weeds (*Taraxacum officinale*, *Erodium cicutarium* and *Tephrosia apollinea*) [11].

The infection of sesame plants by phytoplasmas has been detected through molecular analysis of their 16S rRNA gene, as phytoplasmas are classified within the ‘*Candidatus* Phytoplasma’ species [12] and distinguished in ribosomal groups and subgroups [13] in the same gene. Diverse phytoplasmas were detected in different geographic areas cultivating sesame, in particular, strains in ribosomal groups or subgroups 16SrI (aster yellows) were detected in India [14], 16SrI-B in Myanmar [15], 16SrII-D in Oman [16], and 16SrVI-A and 16SrIX in Türkiye [17,18]. In this latter country, 16SrIX-C and 16SrII-D group phytoplasmas were also identified infecting sesame in the same fields and shown to be transmitted by *Orosius orientalis* [19,20]. However, the majority of the sesame phytoplasmas identified, and for which sequences were deposited in GenBank, belong to the 16SrII group.

Research on phytoplasmas in Iran began 34 years ago, when scientists first discovered that sesame phyllody was associated with the presence of phytoplasmas [21]. Since then, various phytoplasmas have been reported in association with diseases affecting a wide range of host species across around 26 botanical families. The volume of reports concerning the prevalence of these diseases is increasing in Iran, including both diverse phytoplasma strains and a wide range of plant species they infect. To date, phytoplasmas identified in a large number of plant species in Iran belong to nine ribosomal groups, including 16SrI, 16SrII, 16SrIII, 16SrVI, 16SrVII, 16SrIX, 16SrX, 16SrXII, 16SrXIV, and 16SrXXX [22]. Phytoplasmas in 16SrII, 16SrVI, 16SrIX, and 16SrXII groups are the most reported from a broad range of plant species. Phytoplasmas belonging to the 16SrII group are particularly common in the southern region of the country, whereas phytoplasmas from the other three groups are found over a broader geographical area [21]. Some of the phytoplasma diseases in Iran, such as almond and lime witches’ broom, together with grapevine yellows, are highly destructive and have a significant socioeconomic impact [23]. 

Phylogenetic analysis of 16S rRNA gene sequences of detected phytoplasmas indicated the presence of 16SrII-D, 16SrVI-A, and 16SrIX-C phytoplasma strains infecting sesame in the central regions of Iran [24,25]. These phytoplasmas phylogenetically clustered with those previously reported from the eastern and southern regions of the country in the same crop species [26]. The western region of Iran has a favorable climate for the cultivation of the high-value sesame crop, but it is also suitable for the spread of phytoplasmas and their insect vectors; therefore, there is a potential risk of outbreaks in this region. However, no study has been conducted on this pathogen in sesame fields in the Lorestan region to date. This study aims to identify and biologically characterize phytoplasma strains infecting sesame plants in this region to investigate the presence and diversity of phytoplasmas and fill this information gap, contributing also to effective disease management for the mitigation of economic losses.

## 2. Materials and Methods

### 2.1. Sample Collection

**Figure 1:**
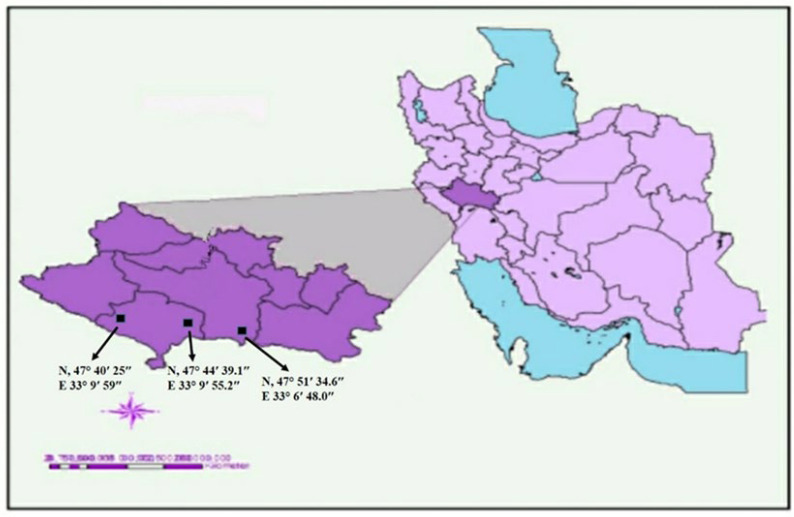
Map of the Lorestan region in Iran with the latitude and longitude of sampled areas for the sesame phyllody disease.

In the summer of 2023, during a field survey of the sesame crops in the Lorestan province region, three locations were selected for sampling at the GPS N 47°51′34.6–E 33°6′48.0; N 47°44′39.1–E 33°9′55.2; and N 47°40′25–E 33°9′59 (Figure 1).

In all nine fields examined, two groups of different symptoms were observed, unevenly distributed in patches in each field. One group of plants exhibited severely small and curly leaves, and a bushy appearance, particularly in the top leaves. The other group displayed phyllody, virescence and a shoot-like structure of the ovary. To estimate the prevalence of the disease, the total number of symptomatic sesame plants was counted in 100 m^2^ size portions (between 250 and 450 plants) and was divided by the total number of plants in the sampling areas. The resulting value was expressed as a percentage by multiplying it by 100. Twelve plant samples exhibiting symptoms (four from each location and two from each type of symptom), along with three symptomless plants (one per location) as negative controls, were randomly collected, placed in individual bags, and transported to the laboratory, where they were stored at 4 °C for short-term use and at −20 °C for long-term preservation. One symptomatic plant from each group of symptoms and from each locality was selected as representative and transferred to the greenhouse with its root system intact to serve as a source of the pathogen for transmission studies.

### 2.2. Transmission of the Pathogen Under Greenhouse Conditions

The pathogen was transmitted to periwinkle [*Catharanthus roseus* (L.) G. Don] plants under an insect-proof greenhouse using dodder (*Cuscuta campestris* L.). To prepare healthy stems of *C. campestris*, seeds were germinated on moist paper, and the resulting seedlings were transferred to five healthy periwinkle plants that had previously tested negative for phytoplasma presence using the nested PCR assays as described below. Once established, the dodder stems were connected to the sesame plants exhibiting different symptoms. Two to four weeks after establishment, the dodder stems from each symptomatic sesame plant were reattached to three healthy periwinkle seedlings. In total, 18 periwinkle plants were used (three plants for each type of symptomatic sesame plant from each locality). This connection was maintained for 45 days. Three healthy periwinkle plants were attached to dodders that were not connected to symptomatic sesame plants to serve as controls, together with three healthy and non-dodder-connected periwinkle plants. The periwinkle plants were kept in 65% garden soil mixed with 35% sandy soil in 30 cm diameter pots and maintained at 24 °C and 60% relative humidity, with a 16 h light and 8 h dark cycle under an insect-proof environment to observe the development of symptoms [27].

### 2.3. DNA Extraction and Polymerase Chain Reaction

DNA extraction was performed using a cetyltrimethylammonium bromide (CTAB) method [28] from the midribs of sesame leaves with small yellow leaves and phyllody symptoms, and from symptomless plants as a negative control. DNA was also extracted from the leaves of periwinkle plants inoculated with dodder and from those used as controls to confirm the transmission by phytoplasma detection and identification.

Detection of phytoplasmas was conducted using nested PCR with the universal primer pairs P1/P7 [29,30] and R16mF2/R16mR1 [31]. PCR was performed using 2x PCR Bio Taq Mix Red (PCRBioSystems Company, London, UK), 100 ng of template DNA, and the primers P1 and P7 at a concentration of 5 pmol/μL in a final volume of 25 μL. The reaction temperature profile consisted of 94 °C for 2 min as initial denaturation, followed by 35 cycles of 94 °C for 30 s, 55 °C for 90 s, 72 °C for 2 min, and a final cycle of 7 min at 72 °C as elongation. The product of this step was diluted 1:30 with sterile distilled water, and 1 μL of this dilution was used as template DNA with primers R16mF2/R16mR1 in nested PCR under the same temperature conditions as before, but with the lower annealing temperature of 50 °C. DNA safe stain (Sinaclon Company, Tehran, Iran) was used to stain the nested PCR products, and 1% agarose gel electrophoresis was performed using a 100 bp as molecular marker (Sinaclon Company, Iran) for their visualization under UV light.

### 2.4. Sequencing, Phylogenetic and Virtual Restriction Fragment Length Polymorphism Analyses

The nested PCR products of three amplicons obtained from each of the two types of sesame symptomatic plants, as well as from symptomatic periwinkle plants after dodder inoculation, were sent to Macrogen (Seoul, Republic of Korea) for purification and sequencing bidirectionally with R16mF2/R16mR1 primer pairs. All the obtained forward and reverse sequences of each strain were converted into a consensus sequence using Geneious Prime 2019.1.3 and with the *i*PhyClassifier tool (http://plantpathology.ba.ars.usda.gov/cgi-bin/resource/iphyclassifier.cgi URL) (accessed on 20 January 2026). They were virtually digested with 17 restriction enzymes, including: *Hae*III, *EcoR*I, *Dra*I, *BstU*I, *Bfa*I, *BamH*I, *Alu*I, *Mse*I, *Sau3A*I, *Kpn*I, *Hpa*II, *Hpa*I, *Hinf*I, *Hha*I, *Taq*I, *Ssp*I and *Rs*aI. The banding patterns were compared to reference profiles available in this database to determine the phytoplasma ribosomal group and subgroup detected. Phylogenetic analysis of the obtained sequences and of other related phytoplasmas was performed using MEGA11 software version 11, employing both the neighbor-joining and the maximum likelihood methods with 1000 bootstrap replicates to evaluate clustering confidence. *Acholeplasma laidlawii* was used as an outgroup in the analysis. BLAST version 2.17.0 comparison with specific reference strains was performed for the identification of the detected ‘*Ca*. Phytoplasma’ species [12].

## 3. Results

### 3.1. Sample Collection

**Figure 2:**
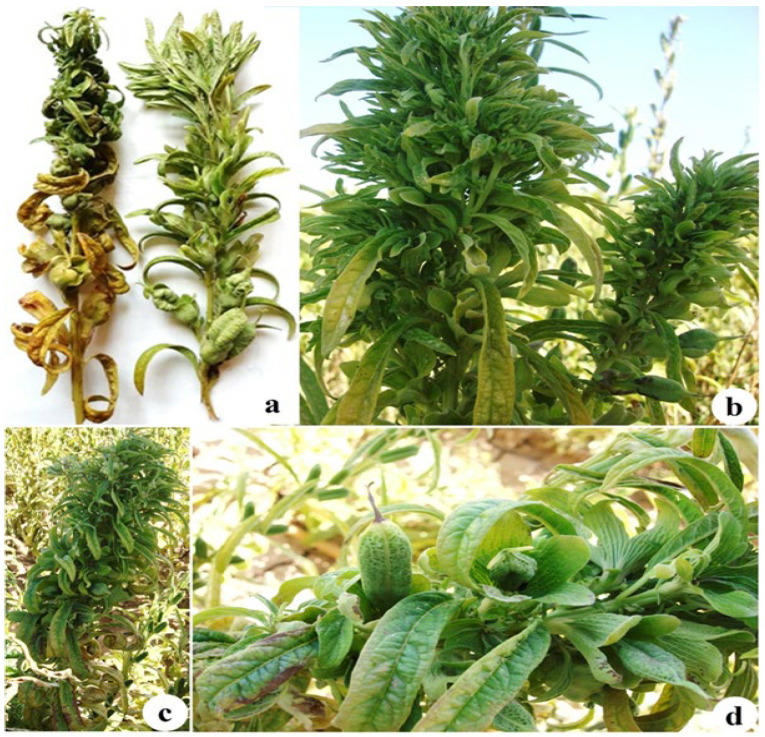
The two types of symptoms in sesame fields: chlorosis, severe curly leaves and twisting, especially in the top leaves, witches’ broom, shortened internodes, reduced leaf size, and a bushy growth pattern (**a**–**c**). Phyllody, flower virescence, and shoot-like structure of the ovary (**d**).

The two types of symptoms in the field were leaf curl and twisting, particularly in the upper leaves, along with a bushy growth pattern and phyllody, virescence, and shoot-like structures in the ovary. Additional symptoms included excessive axillary bud proliferation, shortened internodes, reduced leaf size, witches’ broom, leaf chlorosis, and general stunting (Figure 2). The symptomatology observed in the plants exhibiting two different symptoms was mixed and scattered in the fields. The overall disease incidence in the sesame fields of the Lorestan region based on both symptoms observation was approximately 22%.

### 3.2. Transmission of the Pathogen Under Greenhouse Conditions

**Figure 3:**
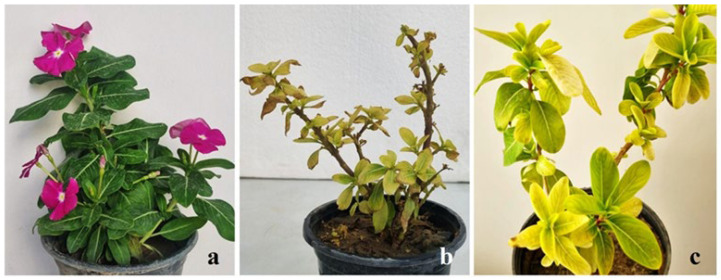
Dodder-inoculated periwinkle plants, exhibiting symptoms a month after inoculation. Healthy control (**a**), reduced leaf size, stunted growth, and phyllody (**b**), and severe chlorosis (**c**).

Four out of the 18 periwinkle plants inoculated with dodder exhibited disease symptoms approximately one month after inoculation. Reduced leaf size, stunted growth, and phyllody were most pronounced in two of the plants, while the remaining plants showed severe chlorosis and no flowering independent of the phytoplasma identified in the sesame source plant used for transmission. In contrast, no symptoms were observed in the healthy periwinkle plants not inoculated with dodder, and also in those in which healthy dodder was attached (Figure 3).

### 3.3. Nested PCR Results

Seven out of the 12 sesame samples exhibiting symptoms of phyllody and leaf curling analyzed by nested PCR using primer pairs P1/P7 and R16mF2/R16mR1 were successfully amplified, producing fragments of 1830 bp and 1434 bp, respectively. In contrast, no amplification was observed in the asymptomatic plants. The nested PCR results using the two mentioned primer pairs were positive for all the samples of the dodder-inoculated periwinkle plants. Nested PCR products, selected based on sample quality from each group of phytoplasma symptoms from sesame and from respective periwinkle dodder-inoculated plants, were sent for purification and sequencing.

### 3.4. Phylogenetic and Virtual RFLP Analyses

The evaluation of the sequences using the BLASTn tool confirmed that they belonged to phytoplasmas. To identify the phytoplasma strains detected, they were compared with nucleotide sequences obtained from the 16S rRNA gene region of phytoplasmas deposited in GenBank among reference strains and phytoplasma-infected sesame and other host plant species in various geographical locations in Iran and abroad (Table 1).

**Figure 4:**
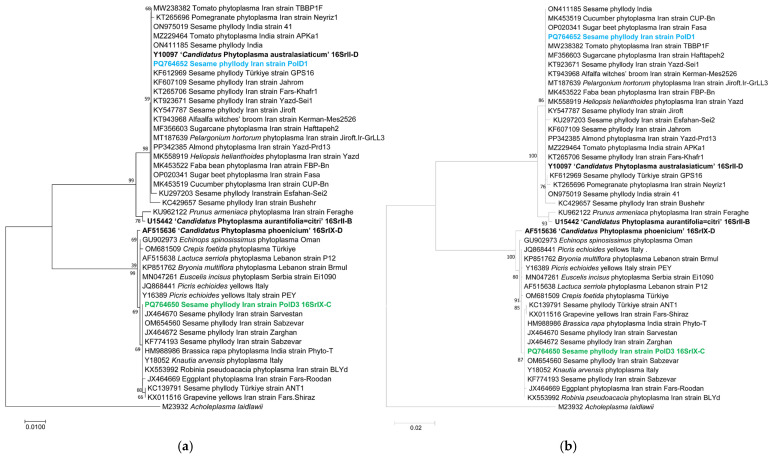
Neighbor-joining trees (1000 bootstraps) constructed using MEGA11 software based on 16S rRNA sequences of the phytoplasma strains used in this study (in blue and green color), and selected phytoplasmas from GenBank (Table 1). In (**a**) the method used is neighbor-joining, while in (**b**) it is maximum likelihood. In bold, reference to ‘*Ca.* Phytoplasma’ strains. *Acholeplasma laidlawii* was used as the outgroup.

Phylogenetic trees were constructed using MEGA11 software with neighbor-joining maximum likelihood methods, with 1000 bootstrap replicates, revealing that the studied strains PolD1 and PolD3 from sesame clustered into two distinct clades referred to ‘*Ca*. P. australasiaticum’ and ‘*Ca*. P. phoenicium’, respectively (Figure 4a,b).

**Figure 5:**
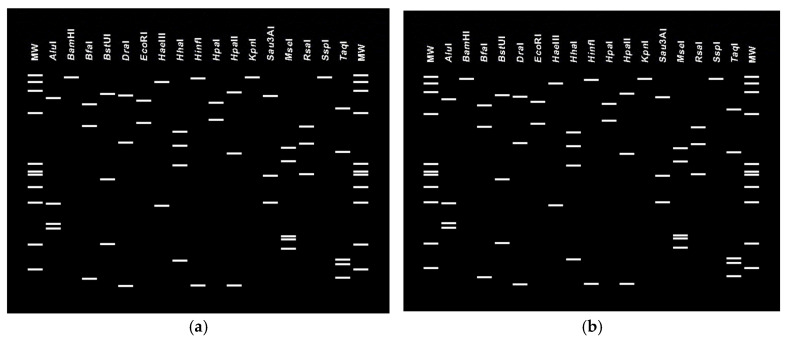
Virtual RFLP profiles generated by *i*PhyClassifier through *in silico* digestion of the 16S rRNA gene F2n/R2 fragments using 17 restriction enzymes. Reference strain of phytoplasma subgroup 16SrIX-C (GenBank accession number Y16389) (**a**). Phytoplasma strain PolD3 from sesame from Iran, enclosed in the subgroup 16SrIX-C (GenBank accession number PQ764650) (**b**).

**Figure 6:**
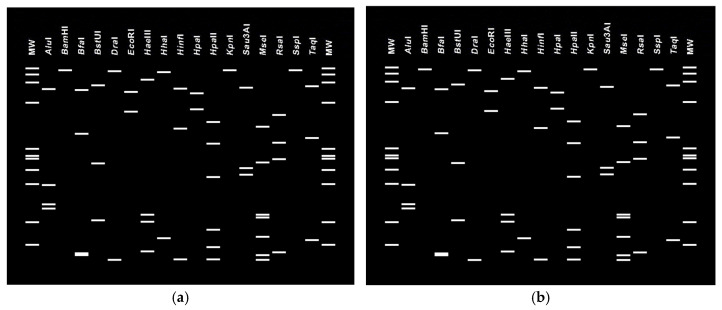
Virtual RFLP profiles generated by *i*PhyClassifier through *in silico* digestion of the 16S rRNA gene F2n/R2 fragments using 17 restriction enzymes. Reference strain of phytoplasma subgroup 16SrII-D ‘*Ca*. P. australasiaticum’ (GenBank accession number Y10097) (**a**). Phytoplasma strain PolD1 from sesame from Iran, enclosed in the 16SrII-D subgroup (GenBank accession number PQ764652) (**b**).

In particular, the analyzed 16S rRNA gene sequence of the phyllody strain PolD3 deposited in GenBank under the accession number PQ764650 exhibited 99.07% identity with ‘*Ca*. P. phoenicium’ (GenBank accession number AF515636), with a strong bootstrap support indicating a close phylogenetic relationship with this reference strain. The PolD3 strain also clustered in the same manner with other strains of ‘*Ca*. P. phoenicium’ from sesame from Fars (Iran) (GenBank accession number JX464670), almond from Lebanon (GenBank accession number AF515636), and *Picris echioides* from Italy (GenBank accession number Y16389). Its virtual RFLP pattern from the 16S rDNA F2nR2 fragment matched with the reference pattern for 16Sr group IX, subgroup C (GenBank accession number Y16389), with a similarity coefficient of 1.00 (Figure 5). Notably, all the restriction enzymes used generated banding patterns with the same sizes and positions in both the PolD3 strain and the reference profiles. These results, along with the findings derived from phylogenetic analyses, validate the subgroup affiliation and the strain identification. The 16S rRNA gene sequence of the strain PolD1 deposited in GenBank under the accession number PQ764652 revealed 100% identity with the ‘*Ca*. P. australasiaticum’ reference strain (GenBank accession number Y10097), and 98.50% with the ‘*Ca*. P. citri’ reference strain associated with witches’ broom disease of lime (WBDL) (GenBank accession number U15442). The virtual RFLP pattern from the 16S rDNA F2n/R2 fragment was the same as the reference profile for 16SrII-D, with a similarity coefficient of 1.00 (GenBank accession number Y10097) (Figure 6). The PolD1 strain clustered with high bootstrap support with the reference strain from papaya from New Zealand (‘*Ca*. P. australasiaticum’) and with a sesame phytoplasma from India (GenBank accession number ON411185).

The sequences of the phytoplasma strains dodder transmitted and obtained from the periwinkle were substantially identical to those detected in the sesame samples and therefore were not deposited in GenBank. It was not possible to distinguish the symptoms in periwinkle associated with the diverse phytoplasma strains present; however, both phytoplasmas resulted in dodder transmission.

## 4. Discussion

The current study confirms the presence of phytoplasmas in several of the tested symptomatic sesame plants; the negative results in some of the samples may be related to a low titer of phytoplasmas, DNA degradation, uneven distribution of the pathogen in the tissues sampled, or a symptom complex of a different nature (abiotic stresses). There was a co-occurrence of phytoplasmas from the 16SrII-D and 16SrIX-C subgroups in different sesame samples from the same fields located in Western Iran. The symptoms associated with the phytoplasmas in the detected subgroups mentioned were consistent with those previously described in sesame in Iran and associated with diverse phytoplasmas [32,33]. As already reported in other phytoplasma disease epidemics, not only on sesame crops, the detection of diverse phytoplasmas did not correspond to differences in symptoms or disease severity in the field. Moreover, it is known that symptomatic observation alone is insufficient to discriminate the diverse agents associated with the disease in sesame that may also be caused by factors other than phytoplasmas. For example, in some growing areas sesame is an important host of *Spiroplasma citri*, and diverse strains of this pathogen that also share the same insect vectors were recently described in Iran [33].

‘*Ca*. P. australasiaticum’ (16SrII-D subgroup) strains are among the prevalent phytoplasmas reported in sesame in Iran, where they are widely associated in sesame with severe symptomatology, particularly in the southern provinces, such as Kerman, Hormozgan, Fars, and Khuzestan [21]. These symptoms result in the loss of seed set especially severe in susceptible sesame genotypes [24]. In sesame, 16SrII-D infections often manifest with shortened internodes, proliferation of axillary buds, witches’ broom, and yellowing of upper leaves, mirroring the symptomatology observed in other hosts such as alfalfa (*Medicago sativa*) infected with the same phytoplasmas [34]. ‘*Ca*. P. australasiaticum’ has a large distribution and has also been reported in ornamental plants, legumes, and weeds in Iran, Oman, Jordan, Egypt, India, China, and Australia [35,36,37,38,39].

On the other hand, ‘*Ca*. P. phoenicium’, has been reported in Iran as associated with yellow symptoms in GF-677 (*Prunus amygdalus* × *P. persica*), wild almond (*P. scoparia*), and in apricots (*P. armeniaca*) [40]. Also, ‘*Ca*. P. phoenicium’ has been reported in stone fruit trees across the Mediterranean and Western Asia, associated with witches’ broom disease. Recent surveys in the Eastern Mediterranean confirmed its presence across multiple hosts, including crops and wild plants in various geographic areas, indicating ecological adaptability and persistence [41].

In this study, the close phylogenetic relationship between the two sesame phytoplasma Lorestan strains and the reference strains was suggested by the strong bootstrap values and the high identity percentages with the respective reference strains. It has been reported that there is genetic diversity among ‘*Ca*. P. phoenicium’ strains, which could be related to different ecological or climatic niches of the phytoplasma populations [42].

The identification of a ‘*Ca*. P. australasiaticum’ (16SrII-D) strain is consistent with reports of this subgroup in several Middle Eastern crops, whereas the detection of 16SrIX-C in sesame fields in Western Iran is notable and may represent an expanding host range for this phytoplasma in this region. This highlights the importance of continuous surveillance to trace pathogen movement and emergence, particularly under changing climatic conditions that may influence insect vector dynamics and facilitate spread into new regions. The co-occurrence of 16SrII-D and 16SrIX-C in symptomatic sesame plants in the same field from the Lorestan region reveals a complex plant disease situation. This highlights the potential for synergistic or antagonistic interactions between strains that could influence symptom severity, diversity, and epidemiologic dynamics. Such variability in symptom expression underscores the importance of detailed and specific diagnostics in phytoplasma research and the development of resistant sesame varieties tailored to diverse pressures from phytoplasma-associated diseases [42]. The detection of a ‘*Ca*. P. australasiaticum’ (16SrII-D) strain in Western Iran in sesame extends its geographic distribution beyond southern Iran, suggesting an adaptive expansion of these phytoplasmas into new agro-ecological zones [21]. Meanwhile, the identification of ‘*Ca*. P. phoenicium’ (16SrIX-C) represents a novel finding in sesame in Western Iran, indicating the involvement of additional phytoplasmas not previously identified infecting this crop in this region of the country, but reported already in other regions [33]. The 16SrIX group has been reported mainly in temperate regions in legumes and ornamentals; however, its presence in sesame suggests either a broader host range or insect vector dynamics that facilitate cross-species transmission [43,44]. The detection of two phytoplasmas, possibly involving diverse insect vector populations, underscores the need for renewed phytoplasma surveillance and insect vector studies in central and western Iran, where *Circulifer haematoceps* is reported to be able to transmit 16SrII-D, 16SrVI-A, and 16SrIX-C phytoplasmas, while *O. albicinctus* is capable of transmitting 16SrII-D strains [24]. Therefore, further studies on the identification of insect vectors present in the surveyed fields will clarify the epidemiological situation in sesame for phyllody disease in the Lorestan province.

The biological and molecular evidence obtained in this study provides new insights into the complexity of sesame phyllody disease in western Iran. The successful detection of both subgroups 16SrII-D and 16SrIX-C from symptomatic sesame plants and their subsequent transmission to periwinkle highlights the coexistence of diverse phytoplasmas in the same agroecosystem. Such simultaneous infections are increasingly recognized in phytoplasma-associated diseases and may have profound implications for disease management.

In this study, molecular analyses based on the F2nR2 region of the 16S rRNA gene, coupled with comparison to virtual RFLP reference profiles, confirmed the presence of two strains of phytoplasmas in subgroups 16SrIX-C and 16SrII-D. The high sequence identity (>99%) and matching RFLP patterns strongly support this classification and suggest further molecular characterization with multilocus analyses. Phylogenetic and virtual RFLP analyses were consistent, grouping all sequences into two distinct clades. The co-existence of divergent phytoplasmas within a single agro-ecological region suggests complex phytoplasma–host–vector interactions and the potential for overlapping disease cycles in neighboring different crops in the same agricultural landscape, since sesame is grown as a monoculture in these areas, and in most cases, phytoplasma-infected tomato fields were extensively cultivated alongside infected sesame fields. It was hypothesized that the shared geographic region of these diverse phytoplasma strains may indicate overlapping transmission cycles and/or vector sharing, as insect vectors such as *Hishimonus phycitis* and *O. albicinctus* are known to transmit phytoplasmas in diverse subgroups across different host species [43].

The presence of two genetically distinct phytoplasmas in the same fields suggests that sesame crops in the region are exposed to multiple inoculum sources, such as different leafhopper vectors, reservoir weed hosts, or perennial alternative hosts. Mixed infections have been shown to enhance disease aggressiveness in several phytoplasma pathosystems, likely through synergistic interactions, competition for host resources, or modulation of host defense pathways. The severe yield losses reported for sesame phyllody, reaching up to 80%, may partly result from such mixed infections, which can intensify hormonal disruption, phyllody induction, and overall plant decline.

From a management perspective, the coexistence of multiple phytoplasma strains presents significant challenges. Phytoplasma diseases lack curative chemical treatments, and insect vector control alone is often insufficient due to the polyphagous nature of many leafhopper vectors and the presence of asymptomatic reservoir hosts in surrounding vegetation. Breeding programs must consider the genetic diversity of phytoplasma populations as resistance effective against one ‘*Ca*. Phytoplasma’ species may not be equally effective against others. Furthermore, understanding the interactions between mixed infections and plant defense responses may guide the selection of genotypes capable of sustaining productivity under complex pathogen pressures. Overall, the documented co-occurrence of 16SrII-D and 16SrIX-C phytoplasmas in western Iran emphasizes the urgent need for integrated disease management programs that combine resistant cultivars, vector monitoring, weed reservoir removal, and molecular surveillance. Continuous monitoring and deeper characterization of local insect vector populations, along with whole genome sequencing of the detected strains, would further clarify transmission dynamics and support the development of robust, region-specific management tools.

The current study is the first confirmation of co-occurring phytoplasmas in 16SrII-D and 16SrIX-C subgroups in different sesame samples from the same fields in Western Iran and reflects the complex ecological interactions in the fields. This finding emphasizes the need for renewed phytoplasma surveillance in Iran improving the understanding of phytoplasma-specific epidemiology in sesame, and the production of disease-resistant varieties.

## Figures and Tables

**Table 1:** Phytoplasma strains used in phylogenetic analyses.

GenBank Accession Number	Host	Strain	Country	Ribosomal Subgroup
JX464670	*Sesamum indicum*	Sarvestan	Iran: Fars	16SrIX-C
JX464669	*Solanum melongena*	Roodan	Iran: Fars	16SrIX-C
HM988986	*Brassica rapa* cv. *toria*	Phyto-T	India	16SrIX-C
Y18052	*Knautia arvensis*	-	Italy	16SrIX-C
JQ868441	*Picris echioides*	PEY	Italy	16SrIX-C
Y16389	*Picris echioides*	PEY	Italy	16SrIX-C
KP851762	*Bryonia multiflora*	Brmul	Lebanon	16SrIX-C
KX011516	*Vitis vinifera*	Shiraz	Iran: Fars	16SrIX-C
JX464672	*Sesamum indicum*	Zarghan	Iran	16SrIX-C
KX553992	*Robinia pseudoacacia*	BLYd	Iran	16SrIX-C
KF774193	*Sesamum indicum*	Sabzevar	Iran: Sabzevar	16SrIX-C
AF515636	*Prunus dulcis*	A4	Lebanon	16SrIX-D
AF515638	*Lactuca serriola*	P12	Lebanon	16SrIX-C
MN047261	*Euscelis incisus*	Ei1090	Serbia	16SrIX-E
GU902973	*Echinops spinosissimus*	-	Oman	16SrIX-J
KC139791	*Sesamum indicum*	ANT1	Türkiye	16SrIX-C
OM681509	*Crepis foetida*	-	Türkiye	16SrIX-C
U15442	*Citrus aurantifolia*	WBDL	Oman	16SrII-B
MK453522	*Vicia faba*	FBP-Bn	Iran	16SrII-D
MK453519	*Cucumis sativus*	CUP-Bn	Iran	16SrII-D
OP020341	*Beta vulgaris*	Fasa	Iran	16SrII-D
KT265696	*Punica granatum*	Neyriz1	Iran	16SrII-D
Y10097	*Carica papaya*	-	New Zealand	16SrII-D
ON411185	*Sesamum radiatum*	-	India	16SrII-D
MZ229464	*Solanum lycopersicum*	APKa1	India	16SrII-D
MW238382	*Solanum lycopersicum*	TBBP1F	Iran	16SrII-D
ON975019	*Sesamum indicum*	41	India	16SrII
KF612969	*Sesamum indicum*	GPS16	Türkiye	16SrII-D
KF607109	*Sesamum indicum*	-	Iran: Darab	16SrII-D
KT265706	*Sesamum indicum*	Khafr1	Iran: Fars	16SrII-D
KU297203	*Sesamum indicum*	Sei2	Iran: Esfahan	16SrII-D
KT923671	*Sesamum indicum*	Sei1	Iran: Yazd	16SrII-D
PP342385	*Prunus dulcis*	Prd13	Iran: Yazd	16SrII-D
MK558919	*Heliopsis helianthoides*	Yazd	Iran: Yazd	16SrII-D
KU962122.2	*Prunus armeniaca*	Feraghe	Iran	16SrII-D
KC429657	*Sesamum indicum*	Bu16	Iran: Bushehr	16SrII-D
KY547787	*Sesamum indicum*	Jiroft	Iran: Kerman	16SrII-D
KT943968	*Medicago sativa*	Mes25, 26	Iran: Zarand	16SrII-D
MF356603	*Saccharum officinalis*	Hafttapeh2	Iran: Hafttapeh	16SrII-D
MT187639	*Pelargonium hortorum*	Ir-GrLL3	Iran: Jiroft	16SrII-D

## Data Availability

The original data presented in the study are openly available from the authors and/or in NCBI GenBank.

## Abbreviations

The following abbreviations are used in this manuscript:

‘*Ca*. P. phoenicium’	‘*Candidatus* Phytoplasma phoenicium’
‘*Ca*. Phytoplasma’	‘*Candidatus* Phytoplasma’
*Ca*. P. australasiaticum’	‘*Candidatus* Phytoplasma australasiaticum’
CTAB	cetyltrimethylammonium bromide
DNA	Deoxyribonucleic acid
GPS	Global Positioning System
*O. albicinctus*	*Orosius albicinctus*
*P. armeniaca*	*Prunus armeniaca*
*P. scoparia*	*Prunus scoparia*
PCR	Polymerase chain reaction
RFLP	Restriction fragment length polymorphism
rRNA gene	Ribosomal nucleic acid
WBDL	Witches’ broom disease of lime
